# Immune Checkpoint-Based Therapies in Colorectal Cancer—Current Approaches and Future Perspectives

**DOI:** 10.3390/ijms27104628

**Published:** 2026-05-21

**Authors:** Katarzyna Nakielska, Jacek Plewka, Marzena Lenart

**Affiliations:** 1 Faculty of Chemistry, Jagiellonian University, Gronostajowa 2, 30-387 Krakow, Poland; katarzyna.nakielska@student.uj.edu.pl (K.N.); jacek.plewka@uj.edu.pl (J.P.); 2 Department of Clinical Immunology, Institute of Pediatrics, Jagiellonian University Medical College, Wielicka 265, 30-663 Krakow, Poland

**Keywords:** immune checkpoints, colorectal cancer, immunotherapy, PD-1, LAG-3, CD161

## Abstract

Colorectal cancer (CRC) is the third most frequently diagnosed malignancy and the second leading cause of cancer-related mortality worldwide, underscoring the need for the development of more effective and durable therapeutic strategies. A key mechanism of tumor immune evasion involves activation of immune checkpoint pathways through the upregulation of inhibitory ligand expression within the tumor microenvironment, leading to lymphocyte exhaustion and impaired antitumor immunity. Consequently, immune checkpoints have emerged as important targets for immunotherapeutic intervention, with significant advances over the past decade. Nevertheless, despite demonstrated clinical benefits in selected patient subpopulations, the overall therapeutic efficacy of immune checkpoint inhibitors remains limited, particularly in the context of CRC. In this review, we provide a comprehensive overview of currently approved immune checkpoint-based immunotherapies for cancer treatment, with a specific focus on CRC, as well as ongoing clinical trials and evolving trends in this area. Furthermore, we discuss emerging targets and novel therapeutic strategies, with particular emphasis on innovative small-molecule inhibitors as potential alternatives to monoclonal antibody-based approaches. Finally, we outline future perspectives and potential directions for advancing immune checkpoint-targeted therapies in CRC.

## 1. Introduction

Colorectal cancer (CRC) is a malignant condition of the last two parts of the large intestine, including the colon and rectum. According to the most recent GLOBOCAN 2022 data from the International Agency for Research on Cancer, colorectal cancer remains the third most commonly diagnosed malignancy and the second leading cause of cancer-related mortality worldwide, with approximately 1.9 million new cases and 900,000 deaths annually [1]. In 2026, an estimated 158,850 new cases of colorectal cancer and 55,230 related deaths are expected in the United States, with a growing proportion of diagnoses (45%) and nearly one-third of deaths occurring in individuals younger than 65 years [2].

Although significant advances have been made in cancer immunotherapy, the identification of novel immune targets and inhibitors remains a major challenge. This is largely because the clinical benefit of immune checkpoint inhibitors is often limited to a subset of patients, while many tumors exhibit primary or acquired resistance. This limitation is particularly evident in colorectal cancer, where only a small fraction of patients responds favorably. Therefore, this review focuses on current trends in immune checkpoint-based immunotherapy for CRC, summarizes ongoing clinical trials and emerging directions, and highlights novel targets, with particular emphasis on innovative inhibitors as alternatives to monoclonal antibody-based treatment.

## 2. Colorectal Cancer

CRC is the third most commonly diagnosed cancer worldwide, ranks second among causes of cancer-related deaths, and is among the cancers with the highest rate of mutations [3]. Early-stage CRC patients are effectively treated with surgical resection and adjuvant chemotherapy. However, patients with advanced disease, particularly those with metastatic CRC, often do not respond successfully to standard treatments [4].

The CRC Subtyping Consortium classified CRC into four consensus molecular subtypes (CMSs) based on distinct molecular and clinical characteristics. CMS1 (14% of cases) is characterized by microsatellite instability, hypermutation, and strong immune activation. CMS2, known as the canonical subtype (37%), is characterized by chromosomal instability, epithelial differentiation, and marked activation of WNT and MYC signaling pathways. CMS3 (13%) is characterized by significant metabolic dysregulation and epithelial features. CMS4 (23%) exhibits prominent activation of transforming growth factor β (TGF-β) signaling, stromal invasion, and angiogenesis and is associated with poorer overall and relapse-free survival [5].

Colorectal carcinogenesis is a heterogeneous disease that has many etiological pathways [6]. One of them is progressive accumulation of genetic alterations over time, which are characterized by a multistep process, ultimately leading to disease progression and metastasis. Among the key signaling pathways involved in CRC initiation and progression, dysregulation of the Wnt/β-catenin pathway plays a central role. This pathway is critically involved in regulating chemoresistance and promoting invasive and metastatic behaviour [7]. In addition to genetic alterations, the tumor microenvironment plays a crucial role in CRC development by facilitating immune evasion and promoting therapeutic resistance [8]. CRC is a heterogeneous disease at both the intertumoral and intratumoral levels, resulting in molecular subgroups with distinct prognoses and responses to therapy [9]. CRC heterogeneity is reflected by four markers, namely microsatellite instability (MSI) or DNA mismatch repair (MMR) deficiency, the CpG island methylator phenotype (CIMP), and somatic mutations in BRAF and KRAS. Microsatellite instability is characterized by a high rate of mutations occurring in short, repetitive DNA sequences known as microsatellites [6]. Molecular genetic studies of CRC have shown that approximately 15% of cases exhibit high microsatellite instability (MSI-H). The MSI-H phenotype is associated with a better prognosis compared to microsatellite-stable tumors (MSS) or those with low microsatellite instability (MSI-L). This more favorable outcome may be partially explained by the pronounced lymphocytic infiltration observed in this subset of cancers. Tumor-infiltrating lymphocytes are observed more frequently in MSI-H tumors than in microsatellite-stable (MSS) tumors (21% vs. 3%) [10,11]. Additionally, a Crohn’s disease—like lymphoid reaction, representing a CRC-specific ectopic lymphoid response—characterized by peritumoral lymphoid aggregates, is commonly seen in MSI-H tumors and is also associated with improved prognosis [12]. Therefore, TILs and CLR may serve as informative prognostic indicators in CRC [13]. Higher levels of TILs within tumor tissue are associated with improved survival rates among cancer patients, including those with colorectal cancer. However, the TIL-induced anti-immune response is counterbalanced by immunosuppressive mechanisms, which may be exploited by tumor cells to escape immune surveillance.

The immune microenvironment in MSI CRC is characterized by a pronounced infiltration of TILs, particularly activated CD8+ cytotoxic T lymphocytes (CTLs) and T helper 1 (Th1) cells that produce interferon-gamma (INF-γ) and express the Th1 transcription factor Tbet. In contrast, these Th1 and CTL immune components are absent in MSS CRC. Consequently, MSI-positive tumors frequently exhibit increased expression of immune-evasive molecules, including immune checkpoint proteins such as programmed cell death protein 1 (PD-1), programmed cell death ligand 1 (PD-L1), cytotoxic T-lymphocyte-associated protein 4 (CTLA-4), lymphocyte activation gene 3 (LAG-3) and indoleamine 2,3-dioxygenase (IDO). Several of these molecules, particularly PD-L1 and IDO, can be induced by IFN-γ. MSI represents a classic example of adaptive resistance. The enhanced expression of immunosuppressive pathways in MSI CRC suggests the presence of an adaptive immune resistance mechanism [14].

CRC heterogeneity extends beyond MSI/MMR status and CMS classification to include histologically aggressive, dedifferentiated phenotypes that are tightly linked to epithelial-mesenchymal transition (EMT) programs and can shape immune responsiveness. In particular, rare poorly cohesive/dedifferentiated variants with plasmacytoid morphology in the gastrointestinal tract (including colorectal localization) show clinicopathological features of high aggressiveness, with frequent lymph node metastases and extremely short overall survival [15]. These tumors display a mesenchymal/EMT-associated immunophenotype characterized by loss of E-cadherin and gain of vimentin, often accompanied by abnormal β-catenin localization, i.e., membrane-to-nuclear translocation or loss, highlighting profound phenotypic plasticity and an invasive program [15]. Such EMT/dedifferentiation-linked states conceptually align with CRC “immune-excluded” behavior, where stromal/ECM barriers and dysregulated trafficking cues restrict cytotoxic lymphocyte penetration and contribute to poor responsiveness to checkpoint blockade in MSS CRC [16]. Importantly, recognizing these aggressive EMT-associated patterns could be based on expression analysis of c-MET and HER2, suggesting a potential targeted-therapy opportunity in a subgroup defined by histologic and EMT features [15].

EMT plays a crucial role in CRC progression. Tumor cells are capable of existing in intermediate or hybrid states that combine both epithelial and mesenchymal characteristics. Both EMT and the hybrid phenotypes significantly contribute to therapeutic resistance, including resistance to immunotherapy, primarily due to cellular phenotypic plasticity rather than a permanent transition to a fully mesenchymal state. Intrinsic cellular plasticity within certain CRC cell populations can give rise to heterogeneity in terms of differentiation status, metastatic capacity, response to therapy and risk of disease recurrence [17]. A hallmark of EMT is the downregulation of E-cadherin expression, which is regulated by a group of EMT transcription factors. In colorectal cancer, a number of microRNAs promote the EMT process by acting, among others, on EMT-related transcription factors. MicroRNAs target transcription factors such as ZEB1, ZEB2, SNAIL, and SLUG. EMT in CRC is associated with an invasive or metastatic phenotype [18,19].

Additionally, tumors with elevated mesenchymal EMT scores show increased PD-L1 expression. Studies of patients treated with immune checkpoint blockade indicate that epithelial-mesenchymal plasticity (EMP) plays a key role in mediating resistance to this therapy. In some patients with urothelial carcinoma characterized by T-cell-infiltrated tumors, strong EMP and stromal characteristics have been associated with reduced responsiveness to the PD-1 inhibitor nivolumab [20,21]. In CRC (particularly MSI tumors), PD-L1 is not primarily expressed by tumor cells but rather by myeloid cells within the TIL microenvironment, which distinguishes CRC from many other tumor types and may influence the response to immunotherapy. Large numbers of PD-L1-positive myeloid cells were observed at the invasive tumor front and within the tumor stroma, and some of them were located between epithelial tumor cells within tumor nests [14].

One of the current therapeutic strategies in CRC treatment involves the use of immune checkpoint inhibitors, which have shown promising results [4]. In CRC, concurrent overexpression of multiple immune checkpoint molecules is frequently observed, with co-expression of two or more markers detected in the majority of cases, particularly in MSI-H tumors compared to MSS tumors [22]. The benefit of immune checkpoint inhibitors is more visible in patients with MSI-H tumors evaluated by MMR protein loss, immunohistochemistry or PCR. Defects in the repair system result in a higher number of somatic mutations, which consequently enhance the tumor immunogenicity [23]. This is caused by frameshift mutations in tumor cells. These mutations create abnormal proteins (frameshift peptides) that the immune system recognizes as foreign, called neoantigens. Because of these neoantigens, tumors with mismatch repair deficiencies are highly responsive to immune checkpoint inhibitors, meaning patients with these tumors often experience strong benefits from immunotherapy [24,25].

Surgical resection remains the primary treatment modality for resectable CRC. For unresectable cases, standard treatment options include chemotherapy, radiotherapy and immunotherapy. However, some of these therapies are limited by poor specificity and cytotoxicity toward healthy cells. Another treatment option is a targeted therapy, which focuses on specific biological molecules involved in cancer growth, helping to inhibit tumor cell growth and division. Targeted therapies use monoclonal antibodies (mAbs) and small-molecule drugs [26,27,28,29]. MAbs used in immunotherapy commonly target immune checkpoints, whereas small molecules have recently emerged as an alternative approach.

Clinical decision-making in CRC is strongly stage- and location-dependent, and for rectal cancer, it additionally hinges on local anatomy, sphincter involvement, and high-quality imaging. In low rectal tumors, sphincter-preserving strategies such as intersphincteric resection (ISR) may be considered in carefully selected patients, and the feasibility of such approaches is guided by preoperative staging (particularly MRI), assessment of surgical planes and mesorectal fascia involvement, and baseline sphincter function (e.g., continence scoring). In a single-center experience with modified ISR for type II/III low rectal tumors, appropriate patient selection combined with standardized preoperative radiotherapy and imaging-based staging enabled favorable long-term oncologic outcomes alongside functional assessment over time, underscoring how local disease management differs from systemic treatment decisions in metastatic CRC. This clinical stratification provides an essential context for immunotherapy: while checkpoint blockade has a defined role primarily in MSI-H/dMMR disease, most MSS/pMMR CRC still relies on multimodal local and systemic strategies, motivating biomarker-guided combinations that address both immune suppression and CRC-specific barriers to response [30].

## 3. Immune Checkpoints Role in Immune Response

Following activation and execution of effector functions, T cells upregulate immune checkpoint molecules that regulate their activity at multiple stages of the immune response, thereby maintaining immune homeostasis. These checkpoints limit excessive immune activation, preserving self-tolerance and protecting against immune-mediated tissue damage [31]. James P. Allison and Tasuku Honjo were awarded the Nobel Prize in Physiology or Medicine 2018 for the discovery of immune checkpoint pathways and their application in immunotherapy [32].

Apart from initially discovered PD-1 and CTLA-4 inhibitory molecules, nowadays, the immune checkpoint family consists of a number of proteins, including LAG-3, T cell immunoglobulin and mucin-domain containing-3 (TIM-3), the T cell immunoglobulin and ITIM domain (TIGIT), the B and T lymphocyte attenuator (BTLA), the V-domain Ig suppressor of T-cell activation (VISTA), CD161, and others [33].

**Figure 1:**
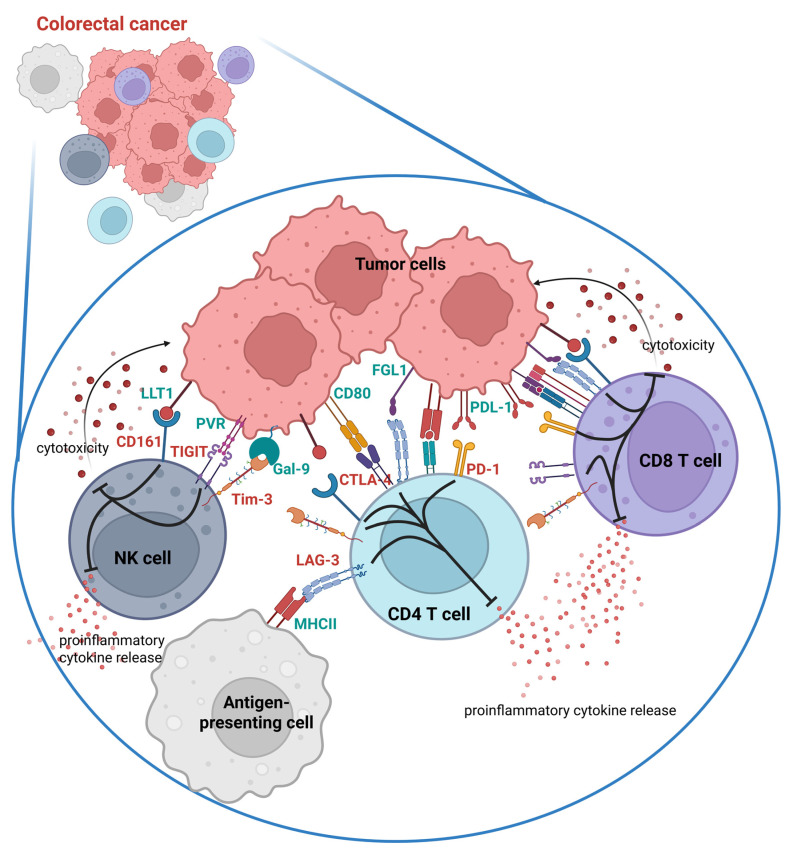
Immune checkpoints and their ligand expression in the CRC tumor microenvironment. The recognition of ligands expressed on tumor cells by the inhibitory receptors results in inhibition of immune cell functions, including proinflammatory cytokine release and cytotoxicity toward tumor cells. Created with BioRender.com.

Modulation of immune checkpoint expression is one of the mechanisms utilized by cancer cells to evade immune response. Tumor cells frequently upregulate immune checkpoint ligands within the tumor microenvironment, thereby promoting T cell exhaustion and immune evasion, while chronic stimulation also induces increased checkpoint receptor expression on immune cells (Figure 1) [34,35,36,37,38,39]. This is why ICs have become an attractive target for immunotherapy.

## 4. Immune Checkpoint Inhibitor-Based Immunotherapy

Currently, FDA-approved immune checkpoint inhibitors target three pathways: PD-1/PD-L1, CTLA-4, and LAG-3. However, the immune checkpoint family comprises a broader range of receptors, including TIM-3, TIGIT, BTLA, HVEM, VISTA, and CD161, many of which are currently under investigation in clinical trials and are gaining increasing attention as potential targets for novel immunotherapies.

To avoid a purely descriptive, “catalog-like” structure, the following sections prioritize CRC-specific clinical relevance and mechanistic insight over exhaustive listing of ligands or trials. For each checkpoint axis, we emphasize the level of evidence in CRC (clinical vs. preclinical), its contribution to resistance in defined CRC subtypes, and its potential role in combination strategies.

### 4.1. PD-1

Programmed death-1 (PD-1) is a transmembrane protein that, together with its ligands, programmed death ligand-1 (PD-L1) and programmed death ligand-2 (PD-L2), belongs to the B7/CD28 family within the immunoglobulin superfamily of receptors [12,40]. PD-1 is widely expressed at the surface of activated T cells, B cells, monocytes, and other immune cells [41]. PD-1/PD-L1 complex constitutes a major immune checkpoint pathway. Under physiological conditions, this pathway plays a critical role in maintaining immune homeostasis, preventing tissue inflammation and autoimmune diseases by inhibiting excessive T-cell activation [12]. PD-L1 is widely expressed in antigen-presenting cells and tissues, such as heart and lung, while the role of PD-L2 is unclear, as it has been reported to exhibit both costimulatory and inhibitory properties [42,43].

PD-L1 and PD-L2 are often expressed on cancer cells, interacting with PD-1 on T cells present in the tumor microenvironment [12]. Cancer cells commonly take advantage of the PD-1 signaling pathway to inhibit T-cell activation, allowing them to evade detection and elimination by the immune system [43]. Therefore, PD-1 constitutes a key regulatory pathway targeted in cancer immunotherapy. This is supported by the observation that PD-L1 and CD80, a CTLA-4 ligand, form cis-heterodimers on the surface of the same cell, which results in the inhibition of PD-L1:PD-1 and CD80:CTLA-4 interactions while preserving CD80-mediated co-stimulation via CD28. In this case, PD-L1 expression on antigen-presenting cells limits CTLA-4-dependent CD80 depletion, implicating the synergy of anti-PD-L1 and anti-CTLA-4 therapy [44]. Inhibition of the PD-1/PD-L1 signaling pathway with therapeutic antibodies reactivates T cells, enhances immune-mediated antitumor activity and facilitates the destruction of cancer cells [45].

The PD-1/PD-L1 immune checkpoint pathway plays an important role in suppressing the immune response in CRC. PD-1 binds to PD-L1 and sends inhibitory signals that reduce T-cell activation and decrease cytokine production. Through PD-1 engagement, its tyrosine residues within the ITIM and ITSM motifs become phosphorylated, creating docking sites for the Src homology 2 (SH2) domain-containing phosphatase SHP-2. Recruitment and activation of SHP-2 initiate an inhibitory signaling cascade by dephosphorylating key proximal TCR and CD28 signaling components, thereby attenuating downstream pathways such as PI3K–AKT and RAS–ERK [46]. These signals ultimately allow cancer cells to evade immune detection and destruction. PD-L1 expression has been associated with advanced disease, metastasis and poor prognosis in CRC [47,48].

In the context of CRC, however, the clinical relevance of this pathway is highly subtype-dependent. Robust and durable responses are largely restricted to MSI-H/dMMR tumors, whereas MSS/pMMR CRC remains predominantly resistant despite PD-1/PD-L1 blockade [49]. This contrast highlights that PD-1 signaling alone is insufficient to overcome immune exclusion and stromal-driven resistance mechanisms characteristic of the majority of CRC cases.

There are seven FDA-approved mAbs targeting PD-1, starting with pembrolizumab in 2014, through nivolumab, cemiplimab, dostarlimab, retifanlimab, and toripalimab, until tislelizumab was accepted in 2024 [50]. The mAbs against PD-L1 include atezolizumab (approved by the FDA in 2016); durvalumab and avelumab (approved in 2017) [51]; and, most recently, cosibelimab, approved in 2024 [52].

Pembrolizumab became the first PD-1 inhibitor approved for patients with advanced or unresectable melanoma who had previously not responded to treatment with ipilimumab and a BRAF inhibitor (if BRAFV600-mutated) based on the findings from the phase I KEYNOTE-001 study. After 6 months, progression-free survival was 45%, and the median overall survival was 25.9 months. Adverse events of grade ≥ 3 were observed [53]. Pembrolizumab indications were further expanded to include first-line treatment of previously untreated advanced melanoma regardless of BRAF mutation status (KEYNOTE-006) [54], as well as a first-line therapy for metastatic non-small-cell lung cancer (NSCLC) with ≥50% PD-L1 expression and without EGFR or ALK genomic tumor aberration (KEYNOTE-024) [55]. Nivolumab is indicated for lymph node-positive or metastatic melanoma following complete resection, advanced renal cell cancer (RCC) after prior anticancer therapy (mTOR inhibitors) and surgically unresectable or metastatic urothelial cancer [56]. Cemiplimab is approved for metastatic or locally advanced cutaneous squamous cell carcinoma in patients who are not candidates for curative surgery or radiation therapy, respectively [57]. One of the first approved mAb targeting PD-L1 was avelumab, which was approved for the treatment of metastatic Merkel cell carcinoma, previously untreated and chemotherapy-resistant. The indication was based on the results of a single-arm phase II JAVELIN trial, which demonstrated an objective response rate of 31.8% (95% CI, 21.9–43.1) [58]. Durvalumab is indicated, among other things, for the treatment of patients with unresectable stage III NSCLC whose disease has not progressed after treatment with chemoradiation, whereas atezolizumab is approved, in combination with carboplatin and etoposide, for the initial treatment of adults with advanced-stage small-cell lung cancer [59].

However, despite the extensive clinical development and broad approval of PD-1/PD-L1 inhibitors across multiple tumor types, their therapeutic efficacy in colorectal cancer remains largely restricted to MSI-H/dMMR tumors, underscoring the limited generalizability of these findings to the majority of CRC patients.

### 4.2. CTLA-4

Cytotoxic T-lymphocyte antigen 4 (CTLA-4) is a member of the immunoglobulin superfamily. It is constitutively expressed by Foxp3+CD4+CD25+ regulatory T (Treg) cells, and its activation is induced in conventional CD4+ and CD8+ T cells [60]. CTLA-4 exhibits a high endocytic capacity and is predominantly localized in intracellular vesicles, with approximately ~90% of the total protein residing intracellularly due to constitutive endocytosis. CTLA-4 is homologous to CD28, yet the latter has an opposite function. CD28 is constitutively expressed on most CD4+ and on a substantial fraction of CD8+ T cells [61]. Both receptors share the ligands CD80 and CD86, which are expressed on the surface of antigen-presenting cells (APCs). However, CTLA-4 binds the ligands with higher affinity and avidity than CD28. Engagement of CTLA-4 inhibits T-cell activation, whereas CD28 interaction with CD80/CD86 provides costimulatory signals in conjunction with T-cell receptor (TCR) signaling [61]. Therefore, CTLA-4 plays a crucial physiological role in suppressing unrestricted cytotoxic T effector cell activity by an indirect reduction of signaling through the co-stimulatory receptor CD28. CTLA-4 outcompetes CD28 for binding to CD80 and CD86, resulting in a reduction in the release of pro-effector cytokines such as IL-12 and cytotoxic enzymes, including perforin and granzyme B. Additionally, CTLA-4 decreases the availability of CD80 and CD86 for CD28 by mediating their endocytosis from APCs. This process increases the activation threshold of T cells, diminishing immune responses to weak antigens such as self-antigens and tumor-associated antigens (TAAs) [62].

Consequently, CTLA-4 has become an important target for immunotherapy aimed at enhancing antitumor immunity. Blocking CTLA-4 with mAbs increases immune responses and promotes antitumor activity [59]. The monoclonal antibody against CTLA-4, ipilimumab, was approved by the FDA in 2011 for the treatment of advanced melanoma [63]. Subsequently, its indication was expanded to include unresectable or metastatic melanoma in adults and pediatric patients (12 years and older) based on an open-label, single-arm trial. Ipilimumab was approved in combined immunotherapy with nivolumab for treatment of previously untreated advanced renal cell carcinoma (RCC) with intermediate or poor risk, regardless of PD-L1 status, based on CheckMate-214, an open-label, randomized (1:1) study [64]. In 2022, the FDA approved the combination of tremelimumab and durvalumab for the treatment of unresectable hepatocellular carcinoma [65]. In 2025, the FDA approved ipilimumab in combination with nivolumab for unresectable or metastatic MSI-H or dMMR CRC. Combined therapy demonstrated superior progression-free survival compared with nivolumab monotherapy across all lines of treatment [66].

Importantly, CTLA-4 blockade has shown clinical benefit in CRC only in combination settings and primarily within the MSI-H/dMMR population [67], reinforcing the need for combinatorial strategies rather than single-pathway inhibition in CRC immunotherapy.

### 4.3. LAG-3

Lymphocyte activation gene-3 (LAG-3) is a type I transmembrane protein that belongs to the immunoglobulin superfamily. It is expressed on the surface of lymphocytes, including CD4 T cells, CD8 T cells, NK cells and Treg cells. LAG-3 is structurally homologous to CD4, as its extracellular domain harbors four Ig-like domains that share about 20% homology with the CD4 receptor [68,69]. LAG-3 binds several ligands, including major histocompatibility complex class II (MHCII), fibrinogen-like protein 1 (FGL-1), galectin-3 (Gal-3), liver and lymph node sinusoidal endothelial cell C-type lectin (LSECtin), α-synuclein preformed fibrils (α-syn PFF) and the T-cell antigen receptor (TCR)-CD3 complex [39,70,71]. MHC-II molecules located on APC are responsible for the presentation of tumor antigens to naïve T cells through binding to the TCR and CD4, inducing T-cell activation. LAG-3 is an inhibitory receptor expressed on tumor-infiltrating lymphocytes (TILs), including CD4^+^ and CD8^+^ T cells and regulatory T cells, where it suppresses T cell activation and function., and it binds to MHCII on APCs with higher affinity than CD4 [70]. As a result, LAG-3 inhibits the binding of CD4 and TCR to MHC-II, thereby inhibiting TCR signaling. In addition, suppression of T-cell activation is mediated by intracellular domains of LAG-3. The inhibitory function of LAG-3 involves its association with the CD3 complex, which impairs T-cell proliferation and cytokine secretion by reducing calcium influx [72]. Upregulation of LAG-3 expression contributes to T-cell exhaustion, thereby promoting tumor immune escape. Moreover, LAG-3 is persistently co-expressed with PD-1 on the exhausted CD8+ T cells within the tumor microenvironment, where these receptors jointly mediate immune evasion by cancer cells [73]. Consequently, in 2022, the FDA approved relatlimab (anti-LAG-3 mAb) in combination with nivolumab (anti-PD-1 mAb) as an immunotherapy for the treatment of melanoma [74].

According to the phase III RELATIVITY-047 trial, dual inhibition of the immune checkpoints LAG-3 and PD-1 resulted in improved progression-free survival compared with PD-1 inhibition alone in patients with previously untreated metastatic or unresectable melanoma. The median progression-free survival was 10.1 months (95% CI, 6.4 to 15.7) with relatlimab–nivolumab as compared with 4.6 months (95% CI, 3.4 to 5.6) with nivolumab [75]. The combination of nivolumab and relatlimab has also been evaluated in patients with resectable clinical stage III or oligometastatic stage IV melanoma in a randomized phase II trial (NCT02519322). In this study, treatment resulted in a 57% pathologic complete response rate and a 70% overall pathologic response rate among the 30 treated patients [76]. What is more, the combination of relatlimab and nivolumab has been evaluated in renal cell carcinoma (NCT02750514), gastric cancer (NCT02996110), gastroesophageal junction adenocarcinoma (NCT03867799) or multiple myeloma (NCT046111269) [77]. However, the clinical utility in CRC is currently uncertain and will likely depend on biomarker-driven patient selection and combination with established checkpoint inhibitors.

### 4.4. TIGIT

T cell immunoreceptor with Ig and ITIM domains (TIGIT) is a receptor belonging to the immunoglobulin superfamily. Its primary role is to negatively regulate both adaptive and innate immune responses. TIGT inhibits the cytotoxicity of effector T and NK cells and enhances the ability of Tregs to suppress effector T cells [78,79,80]. TIGIT is highly overexpressed on tumor-infiltrating lymphocytes (TILs), including CD8+ T cells, CD4+ T cells, Tregs and natural killer (NK) cells, across a wide range of malignancies. TIGIT consists of an extracellular immunoglobulin variable (IgV) domain, a type I transmembrane domain, a highly conserved intracellular inhibitory domain containing an immunoreceptor tyrosine-based inhibitory motif (ITIM), and an immunoglobulin tyrosine tail (ITT) motif [78]. The main ligands of TIGIT are CD155 (PVR), CD112 (PVRL2, nectin-2), CD113 (PVRL3, nectin-3) and nectin-4 (PVRL4), of which CD155 has the highest affinity for this receptor binding. TIGIT competes with other receptors of its family, such as CD226, for binding to CD155. However, CD155 binds TIGIT with higher affinity than CD226, which is an immune-activating receptor. Moreover, TIGIT can inhibit CD226 signaling by disrupting its dimerization on T cells [78,81]. CD155 is overexpressed in various tumors, including NSCLC, melanoma, CRC and glioblastoma [82]. TIGIT suppresses antitumor immune responses at multiple stages of the cancer-immunity cycle and inhibits tumor antigen release by NK cells, impairs T-cell priming by dendritic cells (DCs) and reduces the cytotoxic activity of CD8+ T cells against cancer cells.

TIGIT is commonly co-expressed with PD-1 across multiple T cell subsets. Importantly, PD-1 blockade is associated with approximately a 1.5-fold upregulation of TIGIT on CD8+ T cells [83]. Therefore, dual blockade of TIGIT and PD-1 may represent a promising strategy for effective immunotherapy. Combination immunotherapy with tiragolumab (anti-TIGIT) and atezolizumab (anti-PD-L1) in NSCLC (CITYSCAPE) demonstrated a superior efficacy compared with atezolizumab monotherapy [84]. In contrast, therapy with anti-TIGIT mAbs alone, vibostolimab, and tiragolumab yields no objective responses [85].

Overall, the evidence supporting TIGIT as a therapeutic target in CRC remains limited, with most data derived from preclinical and translational studies. While TIGIT is consistently associated with T-cell exhaustion and immunosuppressive tumor microenvironments, its clinical efficacy in CRC has not yet been established and will likely depend on combination strategies with PD-1/PD-L1 blockade.

### 4.5. TIM-3

T-cell immunoglobulin and mucin-containing molecule-3 (TIM-3) is a type 1 transmembrane molecule, and it is one of the members of the TIM gene family, which includes TIM-1, TIM-3 and TIM-4 [86]. TIM-3 is composed of an immunoglobulin variable (IgV) domain, a mucin stalk domain, a single transmembrane domain, and a cytoplasmic tail domain [87]. TIM-3 interacts with galectin-9 (Gal-9), carcinoembryonic antigen cell adhesion molecule 1 (CEACAM1), high-mobility group protein B1 (HMGB1) and phosphatidylserine (PtdSer). All of these ligands interact with the TIM-3 IgV domain [88]. Interactions of TIM-3 with its ligands play a crucial role in suppressing immune responses, including in cancer and infections. TIM-3 is present on the surface of Th1 T helper cells, monocytes, macrophages, CTLs, NK cells, DCs, Treg cells, Th17 cells, myeloid cells and mast cells [86]. TIM-3 was shown to play a role in activating signaling pathways related to inflammation and tumor metastasis. Gal-9 increases apoptosis in tumor-infiltrating CD8+ T cells and Th1 cells and downregulates their immune function, while TIM-3/CEACAM1 signaling promotes T cell exhaustion [89].

TIM-3 is upregulated in the tumor microenvironment, including CRC. The mouse CRC model proved that TIM-3 plays a role in the apoptosis of CD8+ tumor-infiltrating lymphocytes. CRC patients show a higher frequency of TIM-3+CD8+ T cells in tumor tissues compared with peripheral blood [89]. Preclinical evidence indicates that TIM-3 inhibition can limit tumor progression, notably in combination with PD-1/PD-L1 blockade [90,91]. Currently, TIM-3-targeted immunotherapeutic agents are mainly in phase 1 or 2 clinical trials [92]. One of the phase I clinical trials investigated INCAGN02390, which is a fully human Fc-engineered antibody against TIM-3, in patients with select advanced or metastatic solid tumors, including breast cancer, lung cancer and colorectal cancer. The study results demonstrated modest clinical efficacy and established a recommended dose for phase II investigations of INCAGN02390 in combination with other immunotherapeutic agents for the treatment of advanced cancers [93].

The evidence supporting TIM-3 as a therapeutic target in CRC remains limited and is largely confined to early-stage clinical and preclinical studies. In contrast to TIGIT, its role appears more exploratory, with insufficient data to define its specific contribution to therapeutic response or resistance in CRC.

### 4.6. CD161

CD161 (NKRP1A) is a C-type lectin-like receptor expressed on both NK and T cells and binds LLT1 (CLEC2D, OCIL), its sole known ligand [38,94,95]. CD161 activation results in both T and NK cell exhaustion, which is manifested in a reduction in activation marker expression, cellular cytotoxicity and damped viral infection control [38,96].

The LLT1–CD161 axis inhibits immune cell function in cancer, including CRC. For example, CCR6-CD161+CD57-CD8+ T cells are present in higher frequencies in locally advanced-stage disease (stages II and III) CRC [97]. Moreover, LLT1 overexpression correlates with metastasis and poor prognosis in various cancers. It promotes proliferation and migration and might serve as a poor prognostic factor in, e.g., breast cancer [98], clear cell renal cell carcinoma [99] and CRC [100]. A recently published Cancer Genome Atlas (TCGA) database analysis revealed that LLT1 exhibits high expression in twelve cancers, including CRC, and in three—colon adenocarcinoma (COAD), kidney chromophobe cancer (KICH), and kidney renal clear cell carcinoma (KIRC)—it correlates with poor prognosis. Elevated LLT1 expression correlated with increased infiltration of NK and T cells within the tumor microenvironment, enrichment of exhaustion-related immune biomarkers, and a negative association with pro-inflammatory and tumor-suppressive markers [100].

Both CD161 and LLT1 blocking show promise in cancer immunotherapy. Anti-CD161 mAbs have been shown to inhibit the progression of cancer, such as glioma [38] or hematological malignancies and solid tumors [101]. The most advanced works are on anti-CD161 mAb IMT-009, a monoclonal, Fc-attenuated, fully human IgG1 and a selective LLT1-CD161 complex formation blocker, which is now in a phase 1/2 clinical trial (NCT05565417) for hematological malignancies and solid tumor immunotherapy, including MSS CRC, head and neck cancer, and NSCLC [101]. For LLT1/CLEC2D inhibition, an anti-LLT1 mAb was developed, ZM008, which is now in a phase 1 trial as a single agent or in combination with pembrolizumab in patients with advanced solid tumors (NCT06451497). ZM008 has been proven to activate cytotoxic CD8+ T cells and NK cells, enhancing CD69, perforin/granzyme B expression, and IFNγ/TNFα release, and has led to ~48% tumor growth inhibition with increased immune cell infiltration and apoptotic activity in xenograft models [102].

CD161 remains one of the least characterized immune checkpoint-like pathways in CRC, with evidence largely restricted to preclinical and correlative studies. However, emerging data suggest that the LLT1–CD161 axis may play a more significant role in shaping immune dysfunction within the CRC microenvironment, warranting further investigation.

Taken together, emerging immune checkpoints in CRC are characterized by strong mechanistic rationale but limited clinical validation to date, in contrast to PD-1/PD-L1- and CTLA-4-based strategies. This highlights that future progress will likely depend on rational combination approaches and precise patient stratification rather than the development of additional single-agent therapies.

## 5. Immune Checkpoint-Based Immunotherapy in CRC

Currently, FDA-approved immune checkpoint inhibitor therapies in human cancers, including CRC, target three pathways: PD-1/PD-L1, CTLA-4, and LAG-3 (Table 1).

The first checkpoint inhibitor immunotherapy for CRC that received accelerated approval from the U.S. Food and Drug Administration (FDA) in 2017 [153] was nivolumab, an anti-PD1 mAb. Nivolumab was approved for the treatment of patients aged 12 years and older with dMMR and MSI-H metastatic CRC that had progressed following standard chemotherapy, including fluoropyrimidine, oxaliplatin and irinotecan [124]. Then, in 2020, the FDA also approved pembrolizumab, an anti-PD-1 mAb, for the first-line treatment of patients with unresectable or metastatic MSI-H or dMMR CRC [154].

In 2018, the FDA granted accelerated approval to the combination of ipilimumab, an anti-CTLA-4 mAb, and nivolumab for the treatment of patients ≥ 12 years old with MSI-H or dMMR metastatic CRC that had progressed following treatment with a fluoropyrimidine, oxaliplatin, and irinotecan. Moreover, in 2025, the FDA granted full approval to this combination for the same patient population with unresectable or metastatic MSI-H or dMMR colorectal cancer [155].

Recently, the anti-LAG-3 mAb, favezelimab, along with pembrolizumab (anti-PD-1 mAb), has been evaluated in a phase III clinical trial for PD-L1-positive MSS/pMMR metastatic CRC [156].

Some attempts have been made to develop immunotherapies targeting TIGIT or Tim-3 for colorectal cancer, although most studies remain at the preclinical or phase I stage. For example, a combined blockade of PD-L1 with atezolizumab and TIGIT with tiragolumab restores CD4+ and CD8+ TIL function in a subset of microsatellite-stable colorectal cancers, overcoming resistance to anti-PD-L1 monotherapy and supporting this combination as a potential therapeutic strategy [157]. A recent preclinical study demonstrated that another anti-TIGIT mAb, ociperlimab, enhances antitumor immunity in colon cancer models by inducing ADCC against Tregs, activating NK cells and monocytes, and improving T cell function [157].

In 2025, the Incyte Corporation reported results from a first-in-humans phase I study of the anti-TIM-3 antibody INCAGN02390, demonstrating a favorable safety profile with no dose-limiting toxicities and no maximum tolerated dose reached in heavily pretreated patients. Despite only modest clinical activity, a biologically active dose was selected to support further evaluation in combination immunotherapy strategies for advanced cancers, including colorectal cancer [93].

Taken together, these findings highlight that immune checkpoint inhibition has transformed the therapeutic landscape of CRC, but its efficacy remains largely restricted to biomarker-defined subgroups, particularly MSI-H/dMMR tumors. While dual and emerging multi-checkpoint targeting strategies (e.g., PD-1/CTLA-4 or PD-1/LAG-3 combinations) have demonstrated improved clinical outcomes in selected settings, the majority of MSS/pMMR CRC cases remain refractory to current immunotherapies. Ongoing efforts to target additional immune checkpoints, such as TIGIT and TIM-3, as well as to develop rational combination strategies, aim to overcome these limitations. Ultimately, the integration of molecular stratification, tumor microenvironment profiling, and combinatorial approaches will be critical for expanding the clinical benefit of immunotherapy to a broader population of CRC patients.

## 6. Novel Approaches in CRC Immunotherapy

**Figure 2:**
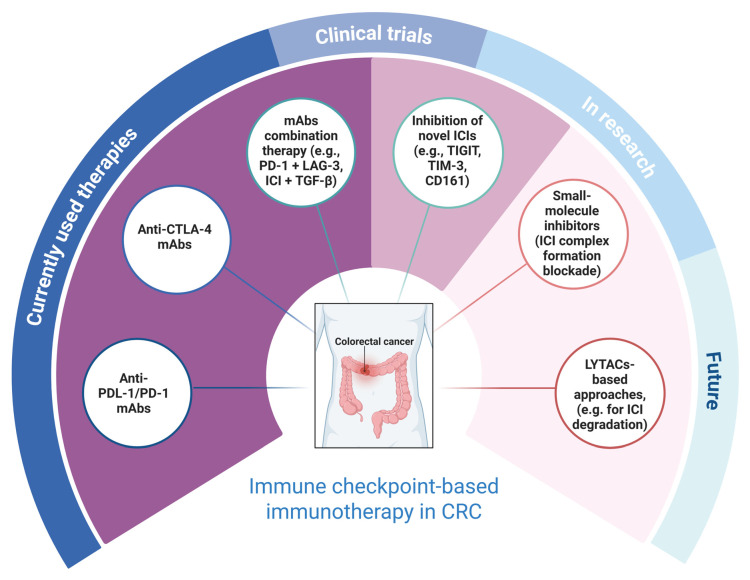
Diagram summarizing ICI-based immunotherapy approaches in CRC treatment, including current, under-development, and future approaches. Created with BioRender.com.

Immune checkpoint inhibitors, such as mAbs, have several limitations, including poor oral bioavailability, immune-related adverse effects and efficacy limited to a subset of cancer patients. One of the types of colorectal cancer, in which ICIs show limited clinical efficacy, is a microsatellite-stable (MSS) colorectal cancer. MSS tumors are characterized by low immunogenicity and, consequently, demonstrate poor responsiveness to ICIs [158]. These factors triggered research into alternative approaches (Figure 2), including small-molecule inhibitors and peptides, as well as novel mAbs combination therapies, not only limited to ICIs but, e.g., also tumor-promoting cytokines such as TGF-β.

This limited immune response is driven by several mechanisms. First, MSS CRC is typically associated with a low mutational burden, associated with reduced neoantigen formation, which limits activation of tumor-specific T cell responses [49]. Additionally, dysregulated chemokine signaling, along with stromal barriers, limits cytotoxic lymphocyte infiltration. Both T cells and NK cells have been shown to exhibit functional impairment or exhaustion [159]. The MSS CRC microenvironment is also enriched in immunosuppressive cell subsets, such as Treg cells and myeloid-derived suppressor cells (MDSC), as well as immunosuppressive cytokines, including TGF-β and IL-10, which further inhibit immune response [160,161].

Finally, resistance to immunotherapy may reflect redundancy within inhibitory signaling pathways, as tumor-infiltrating lymphocytes frequently co-express multiple checkpoint receptors, which act cooperatively to maintain a dysfunctional or exhausted state. Thus, blockade of a single pathway, such as PD-1, often results in compensatory upregulation of alternative inhibitory receptors, limiting restoration of effective antitumor immunoresponse [162]. Together, these mechanisms highlight the need for novel therapeutic strategies targeting additional inhibitory pathways and alternative immune effector cell populations.

In some cancer patients, e.g., in advanced gastric cancer, treated with anti-PD-1 mAb, a rapid progression of the disease can be observed [163]. This phenomenon is associated with the antitumor response of PD-1+ Treg cells. Treg cell functions mostly rely on TGF-β, whose inhibition, in turn, has been shown to overcome PD-1 blockade. For example, in a mouse colon cancer model, anti-GARP:TGF-β1 mAbs, which selectively block a single TGF-β isoform, overcome resistance to PD-1/PD-L1 blockade [164]. Martin et al. developed a selective anti-TGFβ1 antibody, SRK-181, for which coadministration with anti-PD-1 treatment induced profound antitumor responses and survival benefits [165]. Moreover, as TGFβ1 has been shown to increase progression and metastasis of CRC cells, combined immunotherapy against TGFβ1 and immune checkpoints might also potentially improve outcomes for CRC patients.

Overall, resistance to immune checkpoint blockade in CRC is driven by complex immunosuppressive mechanisms, including Treg activity and TGF-β signaling within the tumor microenvironment. Targeting these pathways in combination with PD-1/PD-L1 inhibition represents a promising strategy to enhance therapeutic efficacy and extend clinical benefit beyond MSI-H/dMMR tumors.

### Small-Molecule Inhibitors

Although antibody-based immunotherapies are currently the standard of care for many cancer indications, small-molecule inhibitors (SMIs) and peptides are emerging as attractive alternative modalities for immune checkpoint targeting. Owing to their significantly lower molecular weight, these agents offer several important advantages, including reduced immunogenicity, improved tissue and tumor penetration, and substantially lower manufacturing costs [166]. In contrast to monoclonal antibodies, small molecules are highly amenable to pharmacokinetic optimization, enabling tunable plasma concentrations; shorter and more controllable half-lives; and, in many cases, the possibility of oral administration, which may improve patient compliance and dosing flexibility. Furthermore, their synthetic accessibility supports scalable and cost-effective production.

However, these benefits are accompanied by certain limitations. Small molecules often exhibit shorter systemic persistence, broader biodistribution that may increase the risk of off-target effects, and potentially lower specificity compared to antibodies. In addition, their activity can be species-dependent, posing challenges for the selection of predictive preclinical models [167]. Nevertheless, the overall advantages associated with SMIs and peptides make them a promising and increasingly explored approach, particularly in the context of combination immunotherapies [166].

The most widely studied small-molecule inhibitors for cancer immunotherapy are those targeting PD-1/PD-L1 complex formation [168]. Among these, the orally available PD-L1 inhibitor INCB086550 is one of the most advanced, currently being evaluated in a phase II clinical trial (NCT04629339) in patients with selected solid tumors, including non-small cell lung cancer, urothelial carcinoma, renal cell carcinoma, hepatocellular carcinoma, and melanoma [169]. Additionally, results from a phase I clinical trial (NCT03762447) demonstrated that INCB086550 induces T-cell activation in whole-blood samples from patients with advanced solid tumors, with preliminary evidence of tumor reduction observed in at least one patient [170].

Despite this progress, developing drugs that target PD-L1 or PD-1 remains challenging because these proteins do not possess deep, well-defined binding pockets. Instead, their interaction occurs across a large, hydrophobic, and relatively flat interface of approximately 1700 Å^2^ [169]. Consequently, most reported small-molecule inhibitors targeting PD-L1 are based on substituted biphenyl scaffolds that disrupt PD-1/PD-L1 interaction by inducing PD-L1 dimerization, thereby preventing its engagement with PD-1. This scaffold was originally disclosed by Bristol-Myers Squibb and has served as a foundation for extensive medicinal chemistry optimization [167,171].

Recent progress has expanded well beyond the original biphenyl chemotype, leading to multiple refined structural classes and potency-enhancing strategies. Halogenated biphenyl derivatives improved PD-L1 binding efficiency through halogen-π and hydrophobic interactions, including di-bromo analogues that effectively disrupt the PD-1/PD-L1 interface. In parallel, terphenyl derivatives introduced more rigid and elongated aromatic frameworks capable of stabilizing PD-L1 dimers. Further diversification was achieved through heterocyclic scaffolds, such as imidazopyridine-containing inhibitors, which enhanced shape complementarity and potency, as well as benzylamine derivatives bearing 2-arylmethoxy-substituted dihalogenated biphenyl motifs that demonstrated robust PD-L1 antagonism [169,172,173,174].

A major conceptual advance was the introduction of C_2_-symmetric small-molecule inhibitors, which exhibit highly efficient PD-L1 dimer induction and sub-nanomolar potency in disrupting PD-1/PD-L1 complexes. Clinically relevant representatives include evixapodlin, currently in phase I trials, and INCB086550, which has progressed to phase II [168,170]. These compounds have been validated through comprehensive biochemical and cellular analyses of clinically evaluated PD-L1 inhibitors. Collectively, these developments highlight a rapidly diversifying chemical landscape in PD-L1 SMI research, evolving from early biphenyl backbones toward increasingly sophisticated and potency-optimized scaffolds with clinical potential.

Beyond classical small molecules, macrocyclic peptides have emerged as a complementary and highly promising modality for targeting the PD-1/PD-L1 axis. Owing to their larger surface area and conformational rigidity, macrocycles are particularly well suited to engage with extended, shallow protein–protein interfaces that are otherwise difficult to target with drugs. Early studies demonstrated that macrocyclic peptides identified via display technologies can bind PD-L1 with high affinity and effectively block PD-1/PD-L1 interaction, often mimicking key interfacial hotspots while maintaining favorable selectivity [175]. Subsequent work showed that macrocyclization enhances proteolytic stability, binding affinity, and structural preorganization, enabling potent inhibition in biochemical and cellular assays [175,176]. Structural studies further revealed that some macrocyclic peptides engage PD-L1 differently from small molecules, directly occupying the PD-1 binding surface rather than inducing dimerization.

A prominent example is BMS-986189, a first-generation macrocyclic peptide PD-L1 inhibitor that advanced into a phase I clinical trial (NCT02739373) evaluating safety and pharmacokinetics in healthy volunteers [177]. Although it demonstrated proof-of-concept, its development was limited by suboptimal pharmacokinetics. A second-generation analogue, BMS-986238, was subsequently developed with improved half-life through strategies such as PEGylation or albumin binding. This compound exhibits picomolar binding affinity and potent functional activity comparable to monoclonal antibodies despite its much smaller size. BMS-986238 has completed a phase I clinical study (e.g., NCT06568458), establishing a foundation for further clinical development [178].

In contrast to PD-1/PD-L1, the development of small-molecule or peptide-based inhibitors targeting CTLA-4 remains significantly less advanced. To date, no small-molecule inhibitors of CTLA-4 have entered clinical trials, and most reported compounds target its ligands CD80/CD86 rather than CTLA-4 itself. For example, RhuDex binds CD80 and modulates its interaction with CTLA-4 and CD28 but failed to progress beyond phase II in rheumatoid arthritis. Recent AI-driven approaches have identified the first direct CTLA-4-binding small molecules with micromolar activity and in vivo efficacy; however, these remain at an early preclinical stage [179].

Peptide-based strategies targeting CTLA-4 are also limited to preclinical research. These include linear peptides targeting the MYPPPY loop, helix–loop–helix scaffolds identified through display technologies, and engineered constructs designed to enhance affinity or targeting. While these peptides show activity in vitro and in animal models, none have advanced to clinical evaluation, largely due to limitations in stability, potency, and pharmacokinetics [180].

Among immune checkpoints, LAG-3 represents the youngest target to achieve clinical validation, with its first antibody approved only recently. Despite this progress, non-antibody modalities remain in early development. No small-molecule inhibitors of LAG-3 have entered clinical trials, and current efforts are focused on disrupting interactions with ligands such as MHC class II and FGL1, which involve large, flat binding interfaces similar to PD-1/PD-L1 [181]. Early studies using fragment screening and DNA-encoded libraries have identified small molecules with measurable activity, although their potency remains modest [182].

Peptide-based approaches targeting LAG-3 are also emerging. Phage display and rational design have produced peptides with micromolar to low-micromolar affinity that can partially restore T-cell activation. Early work on macrocyclic and constrained peptides suggests that higher-affinity ligands may be achievable, although these strategies remain at a proof-of-concept stage [183,184]. Overall, in contrast to PD-L1, where both small molecules and macrocyclic peptides have reached clinical evaluation, the LAG-3 field is still in a formative phase, representing an important opportunity for future therapeutic development.

When examined specifically in the context of colorectal cancer, immune checkpoint-based therapies remain highly limited, particularly outside the microsatellite instability-high (MSI H) setting (Table 2). To date, only a single small-molecule immune checkpoint inhibitor has demonstrated a clear clinical signal in CRC: the aforementioned PD-L1 inhibitor INCB086550 developed by Incyte Corporation. In a phase II clinical trial (NCT04629339, later terminated), one patient with MSI-H colorectal cancer achieved a complete response to INCB086550, providing the first clinical proof of concept that small-molecule PD-L1 inhibition can be effective in CRC [185]. A second compound, CA 170, an orally available small molecule claimed to target the PD-L1/VISTA axis, has been evaluated clinically in a phase I study (NCT02812875) and is frequently cited as having CRC relevance [186]. However, serious mechanistic concerns have been raised regarding CA 170, as independent biophysical and functional analyses indicate that it does not directly bind PD-L1, but instead may modulate immune signaling indirectly, calling into question its classification as a direct PD-L1 inhibitor [187]. Beyond these clinical or near-clinical examples, CRC-specific evidence is confined to three compounds validated exclusively in colorectal cancer mouse models. These include the macrocyclic peptide JMPDP 027, which demonstrated robust antitumor activity in the CT26 murine colon carcinoma model [188], as well as the small-molecule PD-L1 inhibitors YPD 30 and its prodrug IMMH 010, both of which showed clear efficacy in the MC38 colorectal cancer model [189]. Notably, all immune checkpoint-directed compounds with documented CRC relevance identified here converge on a single target, PD-L1, underscoring both the centrality of this axis and the paucity of validated small-molecule or peptide modulators of alternative immune checkpoints in CRC. This stands in sharp contrast to the broader CRC drug landscape, where numerous small molecules are used or investigated but act on non-immune checkpoint pathways [29], emphasizing a substantial unmet need and opportunity in CRC immuno-oncology drug discovery.

Overall, small molecules and peptides offer a versatile approach to immune checkpoint modulation, complementing established antibody therapies. PD-L1-targeting inhibitors have progressed the furthest, with several small molecules and macrocyclic peptides demonstrating potent activity and advancing into clinical trials. By contrast, CTLA-4- and LAG-3-directed non-antibody modalities remain largely in discovery or early preclinical stages, with current compounds showing modest in vitro activity and functional effects, underscoring significant opportunities for the development of next-generation immunotherapeutics.

## 7. Future Perspective

Colorectal cancer remains a major clinical challenge, with a substantial proportion of patients diagnosed at advanced stages and five-year survival rates dropping below 15% in metastatic disease, underscoring the urgent need for more effective and durable therapeutic strategies. Future advances in immune checkpoint-targeted therapies for colorectal cancer will likely depend on four interrelated pillars: biomarker-guided patient selection, overcoming resistance in MSS disease, rational checkpoint combinations, and the development of alternative drug modalities beyond monoclonal antibodies. While monotherapies have demonstrated clinical benefit in selected patient populations, their overall efficacy remains limited, especially in microsatellite-stable CRC, where intrinsic resistance mechanisms and compensatory immune suppression pathways play a dominant role. Accordingly, biomarker-driven stratification and rational combination strategies are expected to play a central role in expanding the fraction of patients who benefit from immunotherapy. 

Clinical validation of combinatorial checkpoint blockade has already been achieved with dual targeting of PD-1 and LAG-3. The combination of nivolumab and relatlimab demonstrated improved progression-free survival compared to anti-PD-1 monotherapy, as reported in an FDA-approved therapy for melanoma. Importantly, this concept has also been evaluated directly in colorectal cancer, highlighting both opportunities and limitations. In the phase II CheckMate 142 study, the nivolumab/relatlimab combination produced durable clinical benefit in patients with microsatellite instability-high metastatic colorectal cancer, achieving an objective response rate of 50% and long-lasting responses with a median duration exceeding 40 months [192]. In contrast, a separate phase II trial in mismatch repair-proficient colorectal cancer (NCT03642067) showed limited overall efficacy, although it provided important mechanistic insights and identified potential responsive subgroups [193]. Together, these studies underscore the importance of appropriate biomarker-guided patient selection when deploying rational checkpoint combinations in CRC.

More broadly, a rapidly expanding number of clinical trials are exploring combinations of PD-1/PD-L1 inhibitors with additional immunomodulatory or targeted agents. In colorectal cancer alone, several dozen active and completed studies are investigating PD-1/PD-L1 blockade in combination with other checkpoint inhibitors, cytokine modulators, kinase inhibitors, or epigenetic therapies [194]. This growing clinical effort reflects an emerging consensus that effective immunotherapy will require coordinated modulation of multiple components of the tumor-immune microenvironment rather than reliance on a single pathway.

In this context, small-molecule inhibitors offer unique opportunities for the development of next-generation combination therapies. Their pharmacokinetic flexibility and potential for oral administration make them particularly attractive for multi-target regimens. One emerging strategy involves combining monoclonal antibodies targeting one checkpoint with small molecules acting on a second pathway, thereby leveraging the complementary strengths of both modalities. An alternative and potentially transformative approach is the development of fully small-molecule-based combinations, which could enable oral, cost-effective, and more accessible treatment options, potentially extending to simplified outpatient use.

Recent medicinal chemistry efforts have begun to explore this direction through the design of dual-target small molecules. Examples include compounds that simultaneously modulate PD-L1 and epigenetic regulators such as HDAC6 [195], as well as hybrid molecules targeting PD-L1 together with innate immune pathways such as STING [196]. Similarly, early studies have reported dual inhibitors targeting both PD-L1 and LAG-3, aiming to recapitulate clinically validated antibody combinations within a single chemical entity [197]. Although these approaches remain at a preclinical stage, they highlight a clear trend toward multifunctional immunomodulatory agents capable of coordinated pathway inhibition.

An additional emerging paradigm is the targeted degradation of immune checkpoints rather than their transient inhibition. Building on the success of PROTAC-based approaches, technologies such as LYTACs are being developed to enable the selective removal of extracellular and membrane proteins, including immune receptors [198]. This strategy introduces the possibility of sustained pathway suppression through receptor depletion rather than competitive blockade. The rapid clinical advancement of targeted protein degradation is exemplified by vepdegestrant, the first FDA-approved PROTAC degrader, approved based on the phase III VERITAC-2 trial (NCT05654623) [199]. Although current degraders primarily target intracellular proteins, similar concepts may be extended to immune checkpoints such as PD-L1 or LAG-3, potentially offering more durable and mechanistically distinct therapeutic effects.

Beyond currently established checkpoints, the identification of novel immune regulatory receptors is expected to further expand the therapeutic landscape. Emerging targets such as the LLT1–CD161 axis are gaining attention for their role in tumor immune evasion and may provide additional opportunities for combination strategies. As the field continues to evolve, integrating these newly identified pathways with established PD-1/PD-L1-centered therapies may lead to more effective and durable antitumor responses.

It might also be important to consider NK cells in a deeper way as a therapeutic target, as they, like T lymphocytes, exhibit functional exhaustion within the tumor microenvironment. Current immune checkpoint blockade strategies have been primarily developed to reverse T-cell exhaustion, yet they omit the important role of innate cytotoxic lymphocytes in the antitumor immune response. For example, CTLA-4 is not expressed on NK cells, while PD-1 expression is relatively low and dependent on activation state and microenvironmental factors. Thus, therapies targeting these receptors have limited effects on NK cell functions. Recently, initial approaches focused on targeting NK cell inhibitory receptors, such as NKG2A [200]. However, these strategies focus predominantly on NK cells alone, rather than addressing the coordinated dysfunction of multiple lymphocyte cell subsets. This is why a more effective future strategy may involve targeting immune checkpoints shared by both T and NK cell populations, such as CD161, which represents a promising dual-target receptor enabling simultaneous modulation of adaptive and innate immune responses.

Overall, the future of immune checkpoint modulation in colorectal cancer is likely to be defined by biomarker-guided, rational combination strategies and by the continued expansion of drug modalities beyond antibodies to include small molecules, peptides, and emerging degradation-based approaches. Continued progress will depend on deeper mechanistic understanding, improved biomarker-driven patient selection, and the development of therapeutics capable of precisely and simultaneously modulating complex immune networks.

## Figures and Tables

**Table 1:** Summary of FDA-approved anti-ICIs mAb-based treatment, with indication of time of approval and cancer type application.

mAb Treatment	Year of Approval	Clinical Application	References
PD-1			
Pembrolizumab	2014	Advanced or unresectable melanoma	[53]
	2015	Non-small-cell lung cancer (NSCLC)	[62]
	2017	MSI-H/dMMR solid tumors	[103]
	2018	Head and neck squamous cell carcinoma (HNSCC)	[104]
	2019	Urothelial carcinoma	[105]
	2019	Cervical cancer	[106]
	2019	Esophageal carcinoma	[107]
	2019	Metastatic small-cell lung cancer (SCLC)	[108]
	2020	Tumor mutational burden-high solid tumors	[109]
	2020	Locally recurrent unresectable or metastatic triple-negative breast cancer	[110]
	2021	Primary mediastinal large B cell lymphoma	[111]
	2021	HER2-positive gastric cancer	[112]
	2021	Advanced renal cell cancer (RCC)	[113]
	2021	Cervical cancer	[114]
	2022	MSI-H endometrial cancer	[115]
	2023	Locally advanced unresectable or metastatic biliary tract cancer (BTC)	[116]
	2024	Unresectable advanced or metastatic malignant pleural mesothelioma	[117]
	2025	Muscle-invasive bladder cancer	[118]
Nivolumab	2014	Advanced or unresectable melanoma	[119]
	2015	NSCLC	[120]
	2015	Advanced RCC	[56]
	2016	Classical Hodgkin lymphoma	[121]
	2016	Squamous cell carcinoma of the head and neck	[122]
	2017	Locally advanced or metastatic urothelial carcinoma	[123]
	2017	Metastatic DNA mismatch repair-deficient or MSI-H CRC	[124]
	2017	Hepatocellular carcinoma	[125]
	2021	Urothelial carcinoma	[126]
	2021	Esophageal/gastroesophageal junction cancer	[127]
Cemiplimab	2018	Metastatic or locally advanced cutaneous squamous cell carcinoma	[128]
	2021	NSCLC	[129]
	2021	Locally advanced basal cell carcinoma	[130]
Dostarlimab	2021	dMMR recurrent or advanced endometrial cancer	[131]
Retifanlimab	2023	Metastatic or recurrent locally advanced Merkel cell carcinoma	[132]
	2025	Locally recurrent or metastatic squamous cell carcinoma of the anal canal	[133]
Toripalimab	2023	Nasopharyngeal carcinoma	[134]
Tislelizumab	2024	Esophageal squamous cell carcinoma	[135]
PD-L1			
Durvalumab	2017	Urothelial carcinoma	[136]
	2020	Small-cell lung cancer (SCLC)	[59]
	2022	Biliary tract cancer	[137]
	2025	Resectable gastric or gastroesophageal junction adenocarcinoma	[138]
Atezolizumab	2016	Advanced-stage SCLC	[113]
	2016	NSCLC	[120]
	2016	Urothelial carcinoma	[139]
	2019	Triple-negative breast cancer	[140]
	2020	Unresectable or metastatic melanoma	[141]
	2022	Unresectable or metastatic alveolar soft part sarcoma	[142]
	2025	Extensive-stage SCLC	[143]
Avelumab	2017	Metastatic Merkel cell carcinoma	[59]
	2017	Urothelial carcinoma	[144]
	2019	RCC	[145]
Cosibelimab	2024	Cutaneous squamous cell carcinoma	[52]
CTLA-4			
Ipilimumab	2011	Advanced melanoma	[63]
PD-1 + CTLA-4			
Nivolumab + ipilimumab	2015	Metastatic melanoma	[146]
	2018	Untreated advanced renal cell carcinoma	[64]
	2018	MSI-H/dMMR metastatic CRC	[147]
	2020	Advanced hepatocellular carcinoma	[125]
	2020	Metastatic NSCLC	[148]
	2020	Malignant pleural mesothelioma	[149]
	2021	Metastatic gastric cancer and esophageal adenocarcinoma	[150]
	2025	Unresectable or metastatic MSI-H or dMMR CRC	[66]
	2025	Unresectable or metastatic hepatocellular carcinoma (HCC)	[151]
Tremelimumab + durvalumab	2022	Unresectable hepatocellular carcinoma	[65]
	2022	NSCLC	[65]
PD-1 + LAG-3			
Nivolumab + relatlimab	2022	Unresectable or metastatic melanoma	[74]
Anti-IC mAb + non-IC-based immunotherapy			
Atezolizumab + bevacizumab (anti-VEGF)	2020	Hepatocellular carcinoma	[152]

**Table 2:** Overview of small-molecule-inhibitor-based immune checkpoint therapies in CRC. * direct binding of CA-170 to PD-L1 is not proven.

Tier	Compound	Target/Axis	Modality	CRC Relevance	Clinical Status/Model	Reference
Clinically validated for CRC	INCB086550	PD-L1	Small molecule	One patient with MSI-H colorectal cancer achieved a complete response to INCB086550	NCT04629339 (Ph II, terminated)	[185]
	CA-170 *	PD-L1/VISTA (claimed)	Oral small molecule	Preclinical antitumor efficacy in CRC-relevant models	NCT02812875 (Ph I completed)	[190]
CRC mouse-model validated	JMPDP-027	PD-1/PD-L1	Macrocyclic peptide	CT26 colorectal carcinoma model explicitly validated	CT26 mouse model	[188]
	YPD-30	PD-L1	Small molecule	MC38 CRC model	Preclinical	[191]
	IMMH-010	PD-L1	Small molecule	MC38 CRC model	Preclinical + clinical	[189]

## Data Availability

No new data were created or analyzed in this study. Data sharing is not applicable to this article.
